# Coexistence of adult mosquitoes (Diptera: Culicidae) inside dwellings in a rural community during a dengue outbreak in Sullana, Piura, 2023

**DOI:** 10.17843/rpmesp.2024.411.13416

**Published:** 2024-03-25

**Authors:** Archi Alejandro Ruiz-Polo, Carlos Martín Núñez-Rodríguez, Cindy Yuriko Saavedra-Ríos, Lya Emilia Niño-Mendoza, Rosa Elena Santillan-Valdivia

**Affiliations:** 1 Entomology Research and Training Center, Sub Regional Health Directorate Luciano Castillo Colonna, Piura, Peru Entomology Research and Training Center Sub Regional Health Directorate Luciano Castillo Colonna Piura Peru; 2 Faculty of Biological Sciences, Universidad Nacional Pedro Ruiz Gallo, Lambayeque, Peru. Universidad Nacional Pedro Gallo Faculty of Biological Sciences Universidad Nacional Pedro Ruiz Gallo Lambayeque Peru

To the editor. Although the *Aedes aegypti* (Linnaeus, 1774) mosquito is the species that receives most attention during dengue outbreaks, whether in urban or rural localities, there are other species that go unnoticed and coincide frequently in these epidemiological events. In the context of climate change and its impact on the biological cycle of insects, migration of human populations, mechanical translocation and subsequent adaptation of vectors between endemic and non-endemic areas, and modes of transmission of pathogens ^1^^,^^2^, it is important to consider the potential risk of circulation of more than one etiological agent and co-infection in humans.

These species include *Anopheles albimanus* (Wiedemann, 1821), transmitter of the protozoan parasite *Plasmodium falciparum*, which causes malaria ^3^, *Culex quinquefasciatus* (Say, 1823), transmitter of Rift Valley fever virus, St. Louis encephalitis virus, West Nile virus, filariae and avian malaria parasites ^4^.

Among the mentioned species, attention should be paid to *C. quinquefasciatus*, since recent studies in Brazil have identified it as one of those responsible for transmitting the Zika virus (ZIKV) in recent epidemics, which has been validated by laboratory tests that indicate its potential infection by ZIKV variants that have the ability to replicate in the salivary glands of the mosquito ^5^^,^^6^. Likewise, the relevance of this insect is not only focused on the potential transmission of the ZIKV virus, but also on the subsequent development of diseases that have been associated with infection with the virus, such as microcephaly and Guillain-Barré syndrome in several countries ^7^. In this scientific letter, we present the first report of the coexistence of adult mosquitoes (*Diptera: Culicidae*) inside homes in a rural locality during a dengue outbreak in Sullana, Piura, Peru in 2023.

We conducted a cross-sectional study in the rural town of Marcavelica, located in the province of Sullana, department of Piura. The mosquito trapping schedule was between 4:30 p.m. (twilight) and 9:00 p.m. (night) on June 2, 3, 4, 6 and 8, 2023. A total of 80 dwellings were visited, assessing ten dwellings per day, of which only 50 allowed access. Each house was geo-referenced with the Global Positioning System (GPS).

Entomological captures were carried out following the World Health Organization’s resting capture methodology ^8^, inspecting surfaces of rooms, desks, bathrooms, tables, dining rooms, closets, chairs, furniture, shelves, refrigerators, and other household appliances for 20 minutes. It should be noted that, during mosquito trapping, 240 cases of dengue fever (82 confirmed and 158 probable) were reported in the town of Marcavelica ^9^ between epidemiological weeks 1 to 24 of 2023 (up to June 17).

After capture, the mosquitoes were transferred to the insectary of the Entomology Research and Training Center (CICE), located in the district of Querecotillo. The mosquitoes were exposed to ethyl acetate impregnated in cotton for five minutes, after which time, using entomological tweezers, they were manipulated under a stereoscope for recognition and identification of phenotypic characteristics. Finally, data were tabulated with Microsoft Excel 2021, where the quantity of individuals and their sexual dimorphism at the species level were analyzed.

During the execution of the study, we complied with the following ethical aspects: the consistency and implications of the study were explained to the inhabitants prior to entering each home, so that participation was voluntary and without coercion. Information was handled anonymously, and only for academic purposes, for which we used a data collection instrument validated by experts.

A total of 317 adult mosquitoes were captured from 25 m a.s.l. to 91 m a.s.l., distributed in three genera: *Aedes*, *Culex* and *Anopheles*. The species with the highest population density were *Aedes aegypti* with 201 (63.4%) individuals and *Culex quinquefasciatus* with 114 (36.0%) individuals, with the exception of *Anopheles albimanus* for which only 2 (0.6%) individuals were captured (Table 1).

**Table 1:** Number of individuals per species of captured adult mosquitoes.

Species	Males		Females	
	n	%	n	%
*Aedes aegypti*	64	31.8	137	68.2
*Culex quinquefasciatus*	41	36.0	73	64.0
*Anopheles albimanus*	0	0.0	2	100.0

Regarding sexual dimorphism, we found that 64 (31.8%) *A. aegypti* mosquitoes were male and 137 (68.2%) were female; for *C. quinquefasciatus* 41 (36.0%) were males and 73 (64.0%) were females; and for *A. albimanus* the only 2 captured individuals were females. On the other hand, of the 50 (100.0%) evaluated houses, 45 (90.0%) had more than one inhabitant with dengue symptoms at the time of the entomological captures.

One of the limitations of this study is the sample size, due to the cross-sectional design applied during the dengue outbreak (epidemiological event studied), which prevented us from extending the number of entomological captures due to the risk of infection. Nevertheless, the data obtained are relevant because they show the coexistence of adult mosquitoes (*Diptera: Culicidae*).

In conclusion, our results show that during the dengue outbreak in the rural town of Marcavelica in Sullana, there was coexistence between *Aedes aegypti*, *Culex quinquefasciatus* and *Anopheles albimanus*, the latter in low numbers. This is a relevant finding for future public health interventions in rural areas of Peru, since it is possible to implement and/or propose strategies such as syndromic surveillance of febrile diseases, due to the concomitance of mosquito species that transmit etiological agents.
