# Supplementary material for: Coexistence of adult mosquitoes (Diptera: Culicidae) inside dwellings in a rural community during a dengue outbreak in Sullana, Piura, 2023
Source: Rev Peru Med Exp Salud Publica. 2024 Mar 25;41(1):89–90. doi: 10.17843/rpmesp.2024.411.13416 (PMC11149773; doi:10.17843/rpmesp.2024.411.13416)
Supplement: Translation

# Translation: Coexistencia de mosquitos adultos (Diptera: Culicidae) en el interior de viviendas de una localidad rural durante un brote de dengue en Sullana, Piura, 2023

**Authors:** Archi Alejandro Ruiz-Polo, Carlos Martín Núñez-Rodríguez, Cindy Yuriko Saavedra-Ríos, Lya Emilia Niño-Mendoza, Rosa Elena Santillan-Valdivia

**Affiliations:** 1 Centro de Investigación y Capacitación en Entomología, Dirección Sub Regional de Salud Luciano Castillo Colonna, Piura, Perú.; 2 Facultad de Ciencias Biológicas, Universidad Nacional Pedro Ruiz Gallo, Lambayeque, Perú.

Sr. Editor. Si bien, el mosquito *Aedes aegypti* (Linnaeus, 1774) es la especie que tiene mayor atención durante brotes de dengue, ya sea en una localidad urbana o rural, existen otras especies que pasan desapercibidas coincidiendo con más frecuencia en estos eventos epidemiológicos. En el contexto del cambio climático y su impacto en el ciclo biológico de los insectos, la migración de poblaciones humanas, la translocación mecánica y posterior adaptación de vectores entre áreas endémicas y no endémicas, y los modos de transmisión de los agentes patógenos ^1^^,^^2^, es importante considerar el riesgo potencial de la circulación de más de un agente etiológico y la coinfección en las personas.

Entre estas especies destacan *Anopheles albimanus* (Wiedemann, 1821) transmisor del protozoo parásito *Plasmodium falciparum* causante de la malaria ^3^, *Culex quinquefasciatus* (Say, 1823) transmisor del virus de la fiebre del Valle del Rift, el virus de la encefalitis de San Luis, el virus del Nilo Occidental, las filarias y los parásitos de la malaria aviar ^4^.

Entre las especies mencionadas, se le debe prestar atención a *C. quinquefasciatus;* ya que estudios recientes en Brasil, se ha identificado como uno de los responsables en transmitir el virus del Zika (ZIKV) en epidemias de los últimos años, lo que se ha validado con pruebas de laboratorio que indican su potencial infección por variantes ZIKV que tienen la capacidad de replicarse en las glándulas salivales del mosquito ^5^^,^^6^. Asimismo, la relevancia de este insecto no solo se concentra en la potencial transmisión del virus del ZIKV, sino en el posterior desarrollo de enfermedades que se han asociado a la infección con el virus, como microcefalia y el síndrome de Guillain-Barré en varios países ^7^. En esta carta científica, presentamos el primer reporte de la coexistencia de mosquitos adultos (Diptera: Culicidae) en el interior de viviendas de una localidad rural durante un brote de dengue en Sullana, Piura, Perú en 2023.

Se realizó un estudio transversal en la localidad rural de Marcavelica, ubicada en la provincia de Sullana, departamento de Piura. El horario de captura de los mosquitos fue entre las 4:30 p.m. (crepúsculo) y 9:00 p.m. (noche) en los días 2, 3, 4, 6 y 8 de junio del 2023. Se visitaron 80 viviendas en total, evaluando diez viviendas por día, de las que solo 50 permitieron el ingreso. Cada vivienda fue georreferenciada con el Sistema de Posicionamiento Global (GPS).

Las capturas entomológicas se realizaron siguiendo las indicaciones de la metodología de captura en reposo de la Organización Mundial de la Salud ^8^, inspeccionando superficies de habitaciones, escritorios, baños, mesas, comedor, roperos, sillas, muebles, repisas, refrigeradores y otros artefactos electrodomésticos durante 20 min. Cabe precisar que, durante la captura de mosquitos, entre la semana epidemiológica 1 a la 24 del 2023 (hasta el 17 de junio), en la localidad de Marcavelica se presentaron 240 casos de dengue (82 confirmados y 158 probables) ^9^.

Posterior a la captura, los mosquitos fueron trasladados al insectario del Centro de Investigación y Capacitación en Entomología (CICE), ubicado en el distrito de Querecotillo. Los mosquitos se expusieron a acetato de etilo impregnado en algodón durante cinco minutos, transcurrido el tiempo y haciendo uso de pinzas entomológicas, fueron manipulados bajo estereoscopio para el reconocimiento e identificación de características fenotípicas. Finalmente, los datos se tabularon con Microsoft Excel 2021, donde se analizó la abundancia de individuos y su dimorfismo sexual a nivel de especie.

Durante la ejecución del estudio, se cumplió los siguientes aspectos éticos: previo al ingreso de cada domicilio se explicó su consistencia e implicancia, por lo que la participación fue voluntaria y sin coerción; manejando la información de forma anónima, y ​​únicamente con fines académicos, para lo cual se empleó un instrumento de recolección de datos validado por expertos.

Se capturó un total de 317 ejemplares de mosquitos adultos desde los 25 m s.n.m hasta los 91 m s.n.m, distribuidos en tres géneros: *Aedes, Culex* y *Anopheles.* Las especies con mayor densidad poblacional fueron *Aedes aegypti* con 201 (63,4%) individuos y *Culex quinquefasciatus* con 114 (36,0%) individuos, a excepción de *Anopheles albimanus* que se capturó sólo 2 (0,6%) individuos (Tabla 1).

**Tabla 1:** Número de individuos por especie de mosquitos adultos cap-turados.

Especie	Machos		Hembras	
	n	%	n	%
*Aedes aegypti*	64	31,8	137	68,2
*Culex quinquefasciatus*	41	36,0	73	64,0
*Anopheles albimanus*	0	0,0	2	100,0

En cuanto al dimorfismo sexual, se encontró que en *A. aegypti* 64 (31,8%) fueron machos y 137 (68,2%) fueron hembras; en *C. quinquefasciatus* 41 (36,0%) fueron machos y 73 (64,0%) fueron hembras; y en *A. albimanus* los 2 individuos capturados fueron hembras. Por otro lado, se evidenció que de las 50 (100,0%) viviendas evaluadas, 45 (90,0%) presentaron a más de un habitante con síntomas de dengue en el momento de las capturas entomológicas.

El presente estudio posee la limitación del tamaño muestral, debido a su diseño transversal aplicado durante el brote de dengue (evento epidemiológico estudiado), lo cual impidió extender el número de capturas entomológicas por el riesgo de infección. No obstante, los datos obtenidos son relevantes al evidenciar la coexistencia entre mosquitos adultos (Diptera: Culicidae).

En conclusión, nuestros resultados demuestran que durante el brote de dengue acontecido en la localidad rural de Marcavelica en Sullana; se presentó la coexistencia entre *Aedes aegypti, Culex quinquefasciatus y Anopheles albimanus;* este último en menor número. Un hallazgo relevante para futuras intervenciones de salud pública en localidades rurales del Perú, ya que mediante ello es posible la implementación y/o planteamiento de estrategias como la vigilancia sindrómica de febriles, debido a la concomitancia de especies de mosquitos transmisores de agentes etiológicos.
